# Automatic Production of [^18^F]F-DOPA Using the Raytest SynChrom R&D Module

**DOI:** 10.3390/ph16010010

**Published:** 2022-12-22

**Authors:** Paweł Waśniowski, Jolanta Czuczejko, Michał Chuchra, Mateusz Wędrowski, Dawid Marciniak, Stanisław Sobiak, Bogdan Małkowski

**Affiliations:** 1 Department of Inorganic and Analytical Chemistry, Collegium Medicum in Bydgoszcz, Nicolaus Copernicus University in Torun, ul. Jagiellonska 13-15, 85-067 Bydgoszcz, Poland; 2 Nuclear Medicine Department, Oncology Centre Professor Franciszek Łukaszczyk Memorial, dr I. Romanowskiej 2 Street, 85-796 Bydgoszcz, Poland; 3 Department of Psychiatry, Collegium Medicum in Bydgoszcz, Nicolaus Copernicus University in Torun, ul. Jagiellonska 13-15, 85-067 Bydgoszcz, Poland; 4 Department of Diagnostic Imaging, Collegium Medicum in Bydgoszcz, Nicolaus Copernicus University in Torun, ul. Jagiellonska 13-15, 85-067 Bydgoszcz, Poland; 5 Department of Manufacturing Techniques, Bydgoszcz University of Science and Technology, ul. Kaliskiego 7, 85-796 Bydgoszcz, Poland

**Keywords:** [^18^F]F-DOPA, Raytest SynChrom R&D, radiosynthesis

## Abstract

[^18^F]F-DOPA is widely used in PET diagnostics. Diseases diagnosed with this tracer are schizophrenia, Parkinson’s disease, gliomas, neuroendocrine tumors, pheochromocytomas, and pancreatic adenocarcinoma. It should be noted that the [^18^F]F-DOPA tracer has been known for over 30 years. However, the methods of radiosynthesis applied in the past did not allow its clinical use due to low efficiency and purity. Currently, in the market, one encounters different types of radiosynthesis using the fluorine ^18^F isotope and variants of the same method. The synthesis and its modifications were carried out using a Raytest Synchrom R&D module. The synthesis consists of the following steps: (a) binding of the fluoride anion ^18^F^−^ on an anion exchange column; (b) elution with TBAHCO_3_^−^; (c) nucleophilic fluorination to the ABX 1336 precursor; (d) purification of the intermediate product on the C18ec column; (e) Baeyer–Villiger oxidation; (f) hydrolysis; and (gfinal purification of the crude product on a semipreparative column. The nucleophilic synthesis of [^18^F]F-DOPA was successfully performed in 120 min, using the ABX 1336 precursor on the Raytest SynChrom R&D module, with a radiochemical yield (RCY) of 15%, radiochemical purity (RCP) ≥ 97%, and enantiomeric purity (ee) ≥ 96%.

## 1. Introduction

The 3,4-dihydroxy-L-phenylalanine ([^18^F]F-DOPA) analog, labeled with the fluorine ^18^F isotope, is a tracer used in the imaging of the integrity and function of the dopaminergic nigrostriatal system and neurodegenerative diseases such as Parkinson’s disease via Positron Emission Tomography fused with the Computed Tomography (PET/CT) technique [1,2,3]. This marker has also found applications in oncology, especially in the imaging of neuroendocrine tumors (NETs) [4,5,6] and neuroblastoma [7,8].

In the first clinical applications of [^18^F]F-DOPA, electrophilic methods began to be used with the carrier-added (CA) [^18^F]F_2_ form, using automated synthesis modules [9,10,11,12]. This method, however, presents certain limitations resulting from the restricted access of the centers to the production of [^18^F]F_2_ in the nuclear reaction ^20^Ne(d, α)^18^F, and the system of transporting this gas to the synthesizer. Other limitations include the low molar activity and low efficiency of [^18^F]F-DOPA synthesis [13].

Due to the clinical increase in the need for [^18^F]F-DOPA, several synthesis methods have been developed using the no-carrier-added (NCA) nucleophilic substitution of Fluoride ([^18^F]F^−^) via the ^18^O (p, n) ^18^F nuclear reaction, which solved the above-mentioned problems and allowed for the widespread application of the tracer [14,15,16,17].

The aim of this study was to adapt [^18^F]F-DOPA radiosynthesis using the Baeyer–Villiger oxidation method with the ABX 1336 precursor to the Raytest SynChrom R&D module.

## 2. Results and Discussion

### 2.1. Recovery of the ^18^F Isotope from the Ion Exchange Column

The first stage of the synthesis was the recovery of the fluoride isotope from the bed of the quaternary methyl ammonium (QMA) anion exchange column. Earlier work on the nucleophilic synthesis of [^18^F]F-DOPA reported that both the type and amount of the phase transition catalyst (PTC) have an influence on the formation of by-products, which lowers the yield of the reaction [16,18]. Based on these results, tetrabutylammonium hydrogen carbonate (TBAHCO_3_^−^) was selected as the PTC for the synthesis and a series of elutions of the fluoride ^18^F isotope was performed from the QMA column, using various amounts of TBAHCO_3_^−^. The residues of ^18^F fluoride were confirmed on a QMA column 12 h after elution. The actual amount of the ^18^F residue was then calculated using the half-life formula.

After elution, azeotropic distillation was carried out at 80 °C with an inert gas stream of 800 mbar. The solvent vapors were collected in a vacuum trap immersed in liquid nitrogen. The distillation was repeated twice in order to completely evaporate the water, reducing the reactivity of fluoride. The volume of acetonitrile added to the second azeotropic distillation was always 50 µL higher than the sum of the volumes of TBAHCO_3_^−^ and acetonitrile (MeCN) that remained in the reactor after recovery of the fluoride isotope. The addition of more solvent in the second distillation allows the TBAF[^18^F] complex, remaining from the first azeotropic distillation, to be removed from the walls of the reactor. 

The amount of TBAHCO_3_^−^ and MeCN added during elution from the column influences the activity of ^18^F fluoride collected in the reaction vessel. It was noted that the residues of the ^18^F isotope, with an amount of 100–200 µL TBAHCO_3_^−^, are comparable. The addition of TBAHCO_3_^−^ in an amount of 50 µL results in an average ^18^F fluoride residue of 10,700 MBq. In addition to the amount of ^18^F fluoride recovered, the influence of the time of the first and second azeotropic distillations on the decrease in its activity, caused by drying time, was considered. It was found that the greater amount of reagents added, the longer the evaporation time. Considering the above-mentioned factors, the total decrease in activity was calculated, resulting from the sum of the residual ^18^F fluoride on the column and the decrease in activity due to the time of azeotropic distillation, compared to the starting activity (120 GBq). The most preferable amount of TBAHCO_3_^−^ and MeCN is 100 µL, as a result of the percentage point of activity decrease, which consists of the residual isotope on the ion exchange column and the duration of the azeotropic drying (Table 1).

### 2.2. ^18^F Isotope Labeling of the Precursor

The next phase was to add the precursor dissolved in dimethylformamide (DMF) to the first reactor. Two commercially available amounts of the ABX precursor 1336 were compared, namely, 10 mg and 30 mg. The results are summarized in Table 2.

First, radiolabeling was performed with 10 mg of the precursor in 500 µL of DMF by adding it to the dried TBAF[^18^F] complex. Next, labeling was carried out at 110 °C for 10 min. The radiolabeling efficiency during the first syntheses reached RCY = 40–50%. However, in subsequent syntheses, fluctuations in the labeling efficiency were observed, which decreased to an RCY of 10%. It was noted that the color of the reaction mixture was red at high yields and black at low yields.

**Figure 1:**
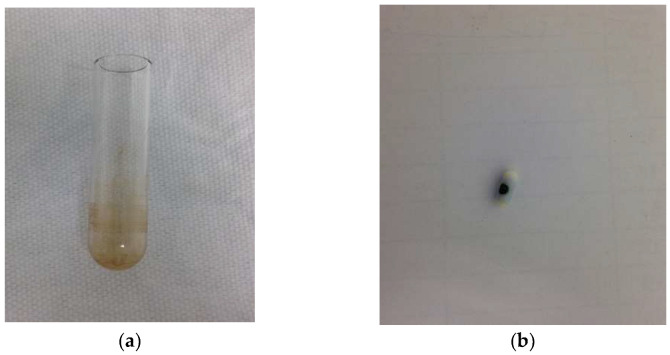
Uncleaned (**a**) reactor and (**b**) magnetic stirrer after the automatic cleaning procedure.

The reactor was dismantled to determine the cause of the labeling fluctuations. It turned out that the liquid remaining after the cleaning procedure was accumulating in the vessel to which the dissolved precursor was added, and under the reactor seal. Additionally, the cleaning procedure did not always clean the reactor, the reactor needle, and the magnetic stirrer of compound residues after radiolabeling (Figure 1a,b).

**Figure 2:**
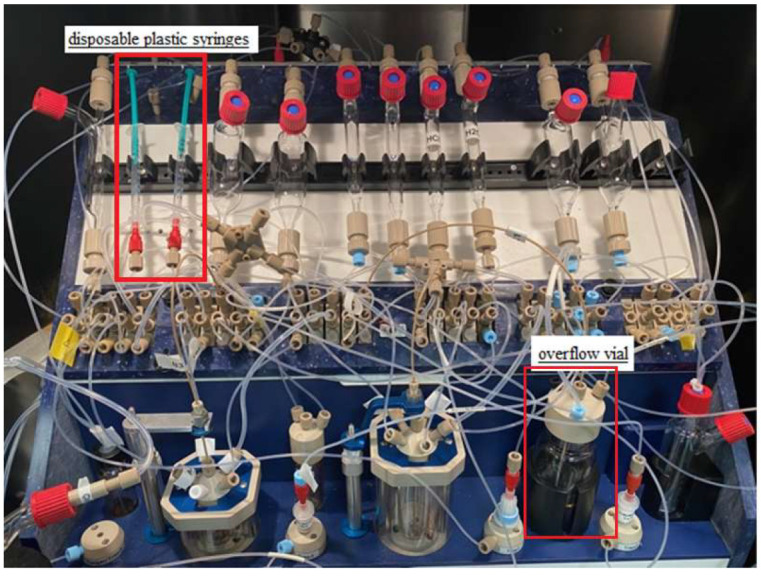
Modifications of the Raytest SynChrom R&D synthesis module.

**Figure 3:**
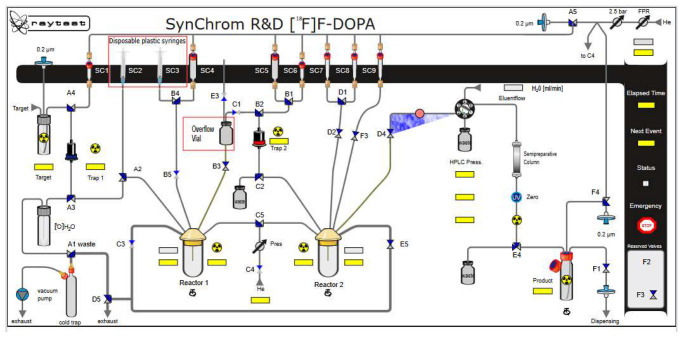
Configuration of the Raytest SynChrom R&D module for [^18^F]F-DOPA synthesis.

Initially, the procedure for cleaning and drying the synthesis module was extended, but an alternative and more practical solution was to replace the precursor and MeCN vessel with a disposable plastic syringe (Figure 2 and Figure 3). Furthermore, manual cleaning of the reactor, magnetic stirrer, needle, and reactor rubber was used. Under these conditions, labeling was performed, which was stable, averaging 68% (see Table 3).

After the nucleophilic isotope exchange reaction, it was necessary to remove the polar solvent and unreacted reagents. For this purpose, various amounts and proportions of solvents were added to the reactor, and the reactor mixture was loaded onto the solid phase extraction (SPE) C18ec purification column.

During the first purification, 4 mL of water was used. This caused the precipitation of organic compounds, leading to blockage of the transfer capillaries and the purification column itself. The amount of 4 mL was due to the capacity of the first reactor. Modifications were made, and an overflow vial (Figure 2 and Figure 3) with 16 mL of water was added to be able to dilute the reactor mixture in more solvent. However, in this case, the transfer capillaries were also blocked.

In the following purification trials, a 4:1 mixture of the two solvents H_2_O/MeCN was applied: First, 4 mL of the mixture from the first reactor, and then an additional 16 mL from the overflow vial, as above. In this case, the transfer lines were not blocked.

Subsequently, radiolabeling was performed with 30 mg of the precursor in a disposable syringe, using an overflow vial and a 4:1 H_2_O/MeCN solvent mixture. A reduction in the flow rate of the reactor mixture to the column was observed during the post-labeling of the reaction mixture to the purification column. The formation of a precipitate was noted. The precipitate likely consisted of the unreacted precursor, insoluble in the solvent mixture used. This led to a low radiochemical yield of approximately 10% (Table 2).

Assessing the results obtained in previous studies on the synthesis of [^18^F]F-DOPA, it was proved in both the studies by Wagner et al. [16] and Pretze et al. [19] that the use of a smaller amount of precursor has a more favorable effect on the synthesis efficiency.

The use of lower amounts of the precursor exerts a favorable influence on the synthesis efficiency. It provides a better effect on the isolation of the intermediate product, the reduction of by-product formation, the specific activity of the product [16], and the substitution of ^18^F, using a combination of K_222_ in DMF instead of TBAHCO_3_^-^ in DMSO [19].

In both of the above-mentioned studies, it was found that the amounts that do not exceed 10 mg of the precursor possess the best influence on the course of the synthesis: Wagner—5, 7 mg (precursor ABX 1335), Pretze—7 mg (precursor ABX 1336). It should be mentioned, however, that after prolonged storage, decomposition of the precursor occurs, which reduces the efficiency of substitution, and in this case, usage in larger amounts allows the efficiency to be maintained at an appropriate level [19].

### 2.3. Oxidation Using mCPBA

Different amounts of m-chloroperoxybenzoic acid (mCBPA) (10 mg, 9 mg, 8 mg, 7 mg, 6 mg, and 5 mg) were taken into account during the tests, and oxidation was carried out at 60 °C for 20 min.

**Figure 4:**
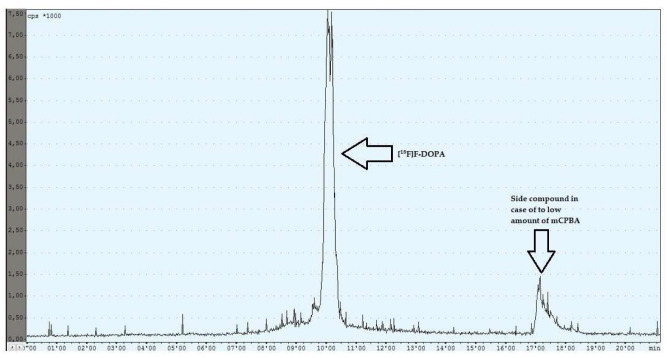
Reaction with too low an amount of mCPBA.

**Figure 5:**
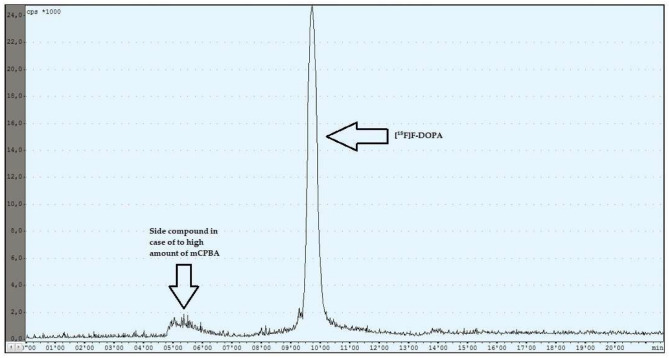
Reaction with too high an amount of mCPBA.

**Figure 6:**
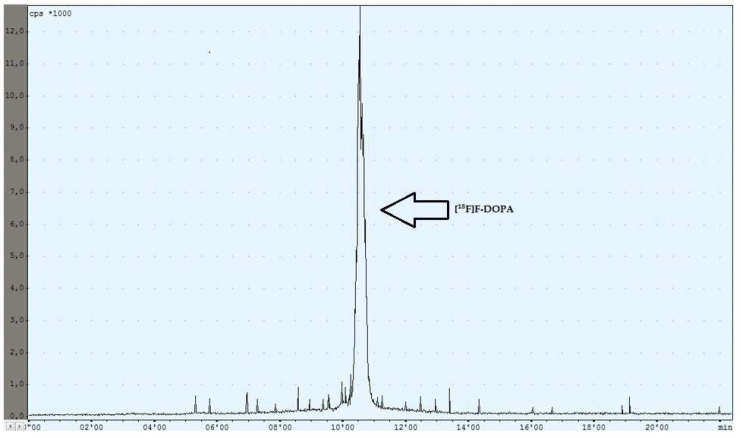
Reaction with the correct amount of mCPBA.

During the experiments with the oxidant, a relationship was observed between the appropriately selected conditions of the oxidation step and the number of individual peaks in the chromatogram, after injecting the crude product into the semipreparative column. In the case of too low an amount of the oxidant used (5 mg, 6 mg), the appearance of the side compound peak was detected, following the specific peak of [^18^F]F-DOPA (Figure 4), whereas in the case of too high an amount of the oxidant (8 mg, 9 mg, 10 mg), the appearance of the side compound peak before the specific peak of [^18^F]F-DOPA was observed (Figure 5). In contrast, when the reaction conditions had been correctly selected (7 mg), no presence of side peaks was found (Figure 6 and Table 4).

The synthesis of [^18^F]F-DOPA remains very sensitive to the amount of oxidant, and to obtain good results, its concentration must be precisely adjusted to achieve the desired reaction conditions. The oxidative power of mCPBA decreases with time, which means that the mCPBA amount used in the synthesis must be constantly adjusted. The selection of appropriate oxidation conditions is also of great importance An appearance and high of the side-compound peaks in the semipreparative column chromatogram may indicate the oxidation condition changes. It provides the operator with the opportunity for a proper reaction.

### 2.4. Hydrolysis

The next stage in the radiosynthesis of [^18^F]F-DOPA was hydrolysis to deprotect groups. In this case, the absence of the imidazolidinone group allows the use of milder hydrolysis conditions compared to the synthesis using the ABX 1335 precursor [15]. As can be seen in Table 5, all compared methods of [^18^F]F-DOPA synthesis are based on the use of milder hydrolysis conditions. Additionally, ethanol (EtOH) was added in an amount of 3%, relative to the amount of 30% hydrochloric acid (HCl).

### 2.5. [^18^F]F-DOPA Crude Product Purification

Purification of the crude final product was carried out using the HPLC method by loading the mixture onto a Hamilton PRP 10 μm 250 × 10 mm semipreparative column. The mobile phase was water and the flow rate was 4 mL/min. The collection of the purified product from the semipreparative column took place by reversing the flow direction of the eluate exiting the column into the product reactor. The proper peak appeared after approximately 10 min (Figure 5). The yield of the total RCY synthesis was 8–10%.

As an alternative, a method using SPE columns was tested. In this case, the reaction mixture was purified using combined C18/HRP columns. The total yield increased to 13–15%. The method of purification using SPE columns was first proposed by Martin et al. [23]. Later, Pretze introduced modifications to this method, using a base to neutralize the acid after hydrolysis. In this work, the Pretze method was used as an alternative for semipreparative purification [19].

By applying the SPE column method, it was possible to eliminate operator error associated with starting the collection of the specific peak too late, which could have reduced the efficiency of the synthesis, or too early, which could have affected the final purity of the product.

## 3. Materials and Methods

### 3.1. General

Chemicals used for the synthesis and quality control of [^18^F]F-DOPA were purchased from Sigma Aldrich. The precursor for the synthesis of [^18^F]F-DOPA (ABX 1336), TBAHCO_3_^−^, and quality control standards was obtained from ABX Advanced Biochemical Compounds GmbH. SPE columns were purchased from Waters and Macherey-Nagel. All radio syntheses and their modifications were performed using the Raytest SynChrom R&D synthesis module.

### 3.2. Cyclotron Production of the Fluoride Isotope ^18^F

The SIEMENS Eclipse 11 MeV Cyclotron was used to produce the ^18^F isotope. The target material to produce ^18^F fluoride was water-enriched in the oxygen isotope [^18^O] H_2_O (2.4 mL) from Cambridge Isotope Laboratories (CIL water ^18^O, 97%). At the end of the bombardment (EOB, t = 0), the amount of the ^18^F fluoride isotope averaged 120 GBq. The ^18^F anion was transported in a 2.4 mL aqueous solution from the cyclotron target to the Raytest SynChrom R&D synthesis module through a Teflon capillary using argon as a pushing gas.

### 3.3. Automatic Radiosynthesis Using the Raytest Synchrom R&D Module

The ^18^F anion, free of impurities reducing its activity, was separated on the Preconditioned Sep-PAK Light QMA Cartridge with CO_3_^2−^ as the counter ions. The fluoride anion was recovered from the QMA column by elution with a 0.075M TBAHCO_3_^−^ solution in MeCN, followed by double azeotropic distillation to remove residual water.

The precursor for the nucleophilic synthesis of [^18^F]F-DOPA (ABX 1336), (S)-3-(5-Formyl-4-methoxymethoxy-2-nitro-phenyl)-2-(trityl-amino)-propionic acid tert-butyl ester in DMF was added to the reaction vessel. The labeling temperature was maintained at 110 °C and the process lasted 10 min.

After the labeling was complete, the water-acetonitrile (H_2_O:MeCN) mixture was added to the reactor, and then the solution was loaded onto a Chromabond C18ec (octadecyl-modified silica, end-capped) purification column. DMF, the free isotope of fluoride [^18^F], 0.075 M TBAHCO_3_^−^ and other contaminants formed during labeling were discharged into the waste. The intermediate was retained on the column, rinsed with 15 mL of H_2_O, and then eluted into a second reactor with 2 mL of MeCN.

**Figure 7:**
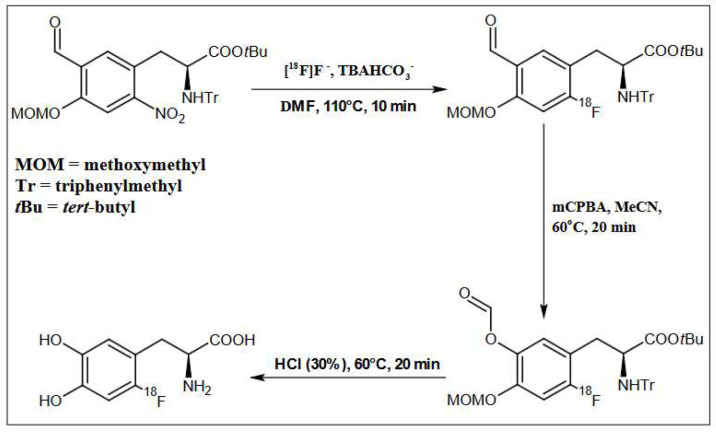
Schematic depiction of reaction conditions for [^18^F]F-DOPA synthesis using the ABX 1336 precursor.

In the next step, oxidation of the intermediate compound using mCBPA was carried out at 60 °C for 16 min. In order to remove the protecting groups from the intermediate compound, 1 mL of 30% HCl containing 3% EtOH was used. Hydrolysis was carried out for 20 min at a temperature of 60 °C. Then the crude [^18^F]F-DOPA was diluted with 5 mL of H_2_O. The last stage was the introduction of the solution on a semipreparative column, Hamilton PRP 10 μm 250 × 10 mm, and in order to purify and collect the final product [^18^F]F-DOPA, the eluent was H_2_O with a flow of 4 mL/min (Figure 3, Figure 7 and Table 6).

### 3.4. Quality Control

General quality control of [^18^F]F-DOPA was performed according to Polish Pharmacopoeia 12th edition (based on the European Pharmacopoeia), the monograph Fluorodopae (18F) ab nucleophila substitutione solutio iniectabilis 04/2019: 2481, and European Pharmacopoeia 10th edition, the monograph Fluorodopae (18F) ab nucleophila substitutione solutio iniectabilis 04/2019: 2481. The estimation of residual solvents was performed according to Polish Pharmacopoeia 12th edition (based on the European Pharmacopoeia), chapter 5.4, and European Pharmacopoeia 10th edition chapter 5.4: Residual solvents.

According to specification and three Quality Control (QC) results (Table 7), QC of the produced [^18^F]F-DOPA included the appearance, potentiometric analysis of pH (4.0–5.5), examination of radionuclide identity (497–526 keV), approximate half-life (105–115 min), radiochemical purity (≥95% of total fluoride-18 radioactivity), enantiomeric purity (L-form ≥ 96% total fluoride-18 radioactivity), chemical purity (0.5 mg/V), residual solvent content (acetonitrile ≤ 0.82 mg/mL, ethanol ≤ 10 mg/mL, methanol ≤ 6 mg/mL, TBA residual (<0.26 mg/mL), analysis of bacterial endotoxin concentration (kinetic chromogenic method; <35 IU/mL), filter integrity test, and assessment of sterility.

The identity and radionuclide purity were tested using the multi-channel analyzer (MUCHA) by Elysia-Raytest.

The radiochemical and chemical purity was verified with a high-performance liquid chromatograph Agilent HPLC 1200 connected in series with a Diode Array Detector (DAD), by means of a Synergi 4P Hydro-RP analytical column, 250 × 4.5 mm; Phenomenex, a flow of 1 mL/min, gradient elution, mobile phase: Aqueous acetic acid (0.1%): methanol 97:3 (*v/v*). To test the enantiomeric identity, an Agilent HPLC 1200 connected in series with a DAD detector (wavelength of 283 nm) was used, using a CHIRAL Daicel CROWNPAK CR (+) 5µ column, 4.0 × 150 mm, at a temperature of 15 °C with isocratic elution, a flow of 0.2 mL/min, and a mobile phase of a 0.02 M aqueous solution of chloric acid (VII).

The TBA residual was examined with an Agilent HPLC 1100 connected in series with the Ultraviolet UV detector (wavelength of 254 nm), using a NUCLEODUR^fi^ C18 Isis analytical column 100 × 4.6 mm 3µm, Macherey–Nagel, with a flow of 0.6 mL/min and a mobile phase of toluenesulfonic acid (0.95 g/L): acetonitrile 25:75 (*v/v*)

The radiochemical purity was also tested by TLC on silica gel plates (aluminum Silica Gel 60 F254 Merck), with a mobile phase of 0.4 M sulfuric (VI) acid: 0.005 M DTPA (1 mL:10 µL DTPA).

A GC gas chromatograph by Agilent with the application of a flame ionization detector (FID) and a Zebron ZB-WAXplus 30m × 0.53 mm × 1.00 µm column was used to test the content of residual solvents. The carrier gas was helium 6.0.

## 4. Conclusions

In conclusion, the nucleophilic method of [^18^F]F-DOPA synthesis was successfully performed using the ABX 1336 precursor and the Raytest SynChrom R&D module. The synthesis was performed for 120 min and the radiochemical yield was 15%, radiochemical purity was ≥97%, and enantiomeric purity was ≥96%.

The application of a smaller amount of the ABX 1336 precursor and the appropriate selection of the mCPBA oxidant in the Baeyer–Villiger oxidation had a better effect on the synthesis radiochemical yield.

The use of SPE purification of the crude product, as compared to purification on a semipreparative column, allowed the elimination of operator error resulting from collecting the specific peak too late, which could reduce the efficiency of the synthesis, or too early, which could affect the final purity of the product. However, the use of SPE purification does not provide the opportunity to observe the changing oxidation conditions and have the appropriate reaction.

It seems that, in order to avoid the above-described problems with the accumulation of impurities after synthesis, a better solution would be to use a module with replaceable disposable radiosynthesis cassettes or a cleaning system immediately after synthesis.

## Figures and Tables

**Table 1:** Effect of the PTC amount and azeotropic distillation time on the activity of the recovered fluoride isotope ^18^F.

Amount of TBAHCO_3_^−^ (µL)	Amount of MeCN (µL)	Activity Remained on the Column 12 H after the End of the Synthesis (MBq)	Calculated Remaining Activity (MBq)	Total Drying Time after Two Azeotropic Distillations (Min)	Percentage Activity Decrease (%)
50 µL	50 µL	1.20 MBq	10,700 MBq	8 min	13.4%
100 µL	100 µL	0.45 MBq	4000 MBq	12 min	10.4%
150 µL	150 µL	0.37 MBq	3300 MBq	16 min	12.1%
200 µL	200 µL	0.25 MBq	2300 MBq	21 min	14.1%
EOB = 120 GBq, 80 °C, *n* = 3					

**Table 2:** Effect of the amount of the precursor on the labeling efficiency.

Amount of Precursor (mg)	Amount of Solvent (µL)	Time (min)	RCY (%)
10 mg	500 µL	10 min	70%
30 mg	1000 µL	10 min	10%
TBAHCO_3_^−^, DMF, 110 °C, *n* = 3			

**Table 3:** Effect of the use of a disposable syringe on labeling efficiency.

	Vial	Syringe
RCY (%)	50%	65%
	20%	70%
	10%	67%
	40%	72%
	15%	65%
TBAHCO_3_^−^, DMF, 10 mg precursor, 110 °C, *n* = 5		

**Table 4:** Comparison of oxidation conditions using mCPBA.

Method	Time (min)	Temperature (°C)	Amount of mCPBA (mg)	Solvent	Amount of Precursor (mg)
Sauvage [20]	20 min	60 °C	-	MeCN	-
Martin [21]	16 min	55 °C	-	MeCN	-
Pretze [19]	20 min	65 °C	5 mg	CH_3_Cl	7 mg
Huang [22]	9.5/5.5 min	65/55 °C	20 mg	MeCN	30 mg
This work	20 min	60 °C	7 mg	MeCN	10 mg

**Table 5:** Comparison of hydrolysis conditions with 30% HCl.

Method	Time (min)	Temperature (°C)
Sauvage [20]	-	40 °C
Martin [21]	20 min	50 °C
Pretze [19]	20 min	65 °C
Huang [22]	20 min	50 °C
This work	20 min	60 °C

**Table 6:** Details of module preparation for automated [^18^F]F-DOPA synthesis.

Vial/Component	Reagent	Volume
SC1	0.075M TBAHCO_3_^−^ in MeCN	100 µL + 100 µL
SC2 syringe	MeCN	250 µL
SC3 syringe	Precursor (ABX 1336) in DMF	10 mg + 500 µL
SC4	MeCN/H_2_O	4 mL (4:1)
OVERFLOW VIAL	MeCN/H_2_O	16 mL (4:1)
SC5	H_2_O	15 mL
SC6	MeCN	2 mL
SC7	mCPBA in MeCN	7 mg + 2 mL
SC8	HCl (30%) + containing 3% EtOH	1 mL + 30 µL
SC9	H_2_O	5 mL
Trap 1	Conditioned QMA	one
Trap 2	Conditioned C18ec	one
Semipreparative HPLC	Hamilton PRP 10 μm 250 × 10 mm H_2_O, flow 4 mL/min	-

**Table 7:** Specification and three QC results of [^18^F]F-DOPA using the Raytest SynChrom R&D module.

QC Test	Specification (Acceptance Criteria)	QC Result		
		QC 1	QC 2	QC 3
Appearance	Clear, colorless solution	Comply	Comply	Comply
pH	4.0–5.5	4.8	5.1	5.0
Radionuclidic identification	Gamma photons have an energy of 497–526 keV	514 keV	516 keV	512 keV
Half life	105–115 min	109.5	109	110
Radiochemical purity (HPLC) 6-[^18^F]-DOPA	≥95% the total radioactivity	99.6%	97.6%	99.4%
Radiochemical purity (TLC) 6-[^18^F]-DOPA	≥95% the total radioactivity	97.9%	96.2%	95.67%
Enantiomeric purity L-form of 6-[^18^F]-DOPA	≥96% the total radioactivity	97.4%	98.9%	98.1%
Chemical purity (HPLC) All chemical impurities Including total peak of 6-fluoro-L-DOPA	2.5 mg/5 mL (0.5 mg/mL)	0.0107 mg/mL	0.018 mg/mL	0.4408 mg/mL
Residual TBA	<1.3 mg/5 mL (<0.26 mg/mL)	<0.26 mg/mL	<0.26 mg/mL	<0.26 mg/mL
Residual solvents:				
Ethanol (GC)	≤50 mg/5 mL (≤10 mg/mL)	1.063 mg/mL	0.004 mg/mL	0.2706 mg/mL
Acetonitrile (GC)	≤ 4.1 mg/5 mL (≤ 0.82 mg/mL)	0.7854 mg/mL	0.0023 mg/mL	0.0029 mg/mL
Methanol (GC)	≤30 mg/5 mL (≤6 mg/mL)	0.8294 mg/mL	0.0028 mg/mL	0.0008 mg/mL
Bacterial endotoxins	<175 EU/5 mL (<35 EU/mL)	<0.250 EU/mL	<0.250 EU/mL	<0.250 EU/mL
Filter integrity test	≥3 Bar	4.0 Bar	4.2 Bar	4.0 Bar
Sterility *	Sterile	Comply	Comply	Comply

* The preparation may be released for use before completion of the test.

## Data Availability

Data is contained within the article.
